# Clinically diverse and perinatally lethal syndromes with urorectal septum malformation sequence

**DOI:** 10.1002/ajmg.a.63067

**Published:** 2022-12-07

**Authors:** Shalini S. Nayak, Robert Harkness, Anju Shukla, Siddharth Banka, William G. Newman, Katta M. Girisha

**Affiliations:** 1 Department of Medical Genetics, Kasturba Medical College, Manipal, Manipal Academy of Higher Education, Manipal, Karnataka, India; 2 Manchester Centre for Genomic Medicine, Manchester University NHS Foundation Trust, Manchester, UK; 3 Evolution, Infection and Genomics, School of Biological Sciences, Faculty of Biology, Medicine and Health, University of Manchester, Manchester, UK; 4 Department of Genetics, College of Medicine and Health Sciences, Sultan Qaboos University, Muscat, Oman

**Keywords:** ambiguous genitalia, imperforate anus, persistent cloaca, urorectal septum malformation sequence

## Abstract

Urorectal septum malformation sequence (URSMS) is characterized by a spectrum of anomalies of the urogenital system, hindgut and perineum. It is presumed to be a constellation of an embryonic defect. Herein, we analyzed the clinically diverse syndromes associated with URSMS in our perinatal evaluation unit. We reviewed fetuses with URSMS in referrals for perinatal autopsy over a period of 3 years. Chromosomal microarray and genome sequencing were performed whenever feasible. Literature was reviewed for syndromes or malformations with URSMS. We ascertained URSMS in 12 of the 215 (5%) fetuses. Nine fetuses (75%) had complete URSMS and remainder had partial/intermediate URSMS. Eleven fetuses had malformations of other systems that included: cerebral ventriculomegaly; right aortic arch with double outlet right ventricle; microcephaly with fetal akinesia deformation sequence; ventricular septal defect and radial ray anomaly; thoraco-abdominoschisis and limb defects; myelomeningocele; spina bifida and fused iliac bones; omphalocele; occipital encephalocele; lower limb amelia and cleft foot. We report on six fetuses with recurrent and five fetuses with unique malformations/patterns where URSMS is a component. Exome sequencing (one family) and genome sequencing (eight families) were performed and were nondiagnostic. Additionally, we review the literature for genetic basis of this condition. URMS is a clinically heterogeneous condition and is a component of several multiple malformation syndromes. We describe several unique and recurrent malformations associated with URSMS.

## 1 Introduction

Urorectal septum malformation sequence (URSMS) refers to a spectrum of anomalies involving urogenital system, hindgut and perineum. It is hypothesized that URSMS results from deficiency of the caudal end of the mesoderm or defect in the cloacal endoderm, peri-cloacal mesenchyme, and genital ectoderm arising during early embryogenesis (Jain et al., 2008). However, a definitive cause is not yet identified. This sequence is described as complete, partial, intermediate, and urogenital sinus anomaly based on the persistent cloaca and perineal openings (Huang et al., 2016; Wheeler & Weaver, 2001).

Often, other malformations are also observed with URSMS (Tennant et al., 2014). Recurrence in families (vertical and horizontal), though extremely rare, has also been reported (Aggarwal & Phadke, 2013; Mills & Pergament, 1997) suggesting the existence of yet undetermined monogenic etiology. Hsu et al. provided the first evidence by identifying de novo variants in Caudal Type Homeo Box Transcription Factor 2 (*CDX2*) in patients with persistent cloaca (Hsu et al., 2018). Herein, we describe 12 fetuses with syndromic and non-syndromic URSMS depicting the clinically diverse perinatal lethal malformations.

## 2 Subjects and Methods

We reviewed fetuses with URSMS in referrals for perinatal autopsy over a period of 3 years at our perinatal genetics services clinic. The study has the approval of the institutional ethics committee and informed consents were taken from the families. Perinatal evaluation was performed (Khong & Malcomson, 2015; Nayak et al., 2015) in all the fetuses. Family history of the couple, antenatal details, exposure to teratogens, consanguinity, three-generation pedigree, findings on antenatal ultrasonography and fetal echocardiography, results of laboratory tests, mode of delivery, and gestational age were systematically documented. Fetal weight, total length, foot length, head circumference, chest circumference, and other measurements were taken and compared with available standard growth charts (Khong & Malcomson, 2015). Anteroposterior and lateral views of the fetus were radiographed to rule out skeletal abnormalities. Additionally, magnetic resonance imaging of the fetal brain/whole body was performed whenever possible. We collected appropriate samples such as skin biopsy, liver, muscle, bone marrow, or umbilical cord from the fetuses. They were used to obtain DNA/RNA/cell lines. An external examination was done from head to toe to document dysmorphism/anomalies. Dissection of the whole body was performed using standard perinatal autopsy procedures (Khong & Malcomson, 2015; Osborn & Lowe, 2017). All internal organs/structures were examined carefully. The normal anatomy and anomalies observed were documented with photographs. Careful examination of placenta was carried out to rule out the cause of fetal demise. Histopathological examination of fetal tissues/organs was performed as appropriate.

Chromosomal microarray, karyotype, exome sequencing, and genome sequencing were performed using fetal genomic DNA whenever possible. For whole genome sequencing (WGS), DNA library preparation and sequencing was performed using DNBSeq platform at Beijing Genomics Institute (BGI), China with standard 30× sequencing depth. Reads were aligned to hg19 human reference genome via Burrows–Wheeler Aligner (BWA v.0.6.2). Variants were called using Genome Analysis Toolkit (GATK) 4.0. Annotation was performed using VarSeqTM v2.2 (Golden Helix Inc., Boseman, MT, USA). Rare variants (<0.01%) were identified from population frequencies observed in the Genome Aggregation Database (gnomAD) and using population frequencies from in-house exome sequencing. In silico variant predictions were modeled using Polyphen-2 (http://genetics.bwh.harvard.edu/pph2/), Mutation taster (http://www.mutationtaster.org/), Mutation Assessor (http://mutationassessor.org/r3/), SIFT (Sorting Intolerant From Tolerant) (http://sift.bii.a-star.edu.sg/), CADD (GRCh37-v1.4), and REVEL (Ioannidis et al., 2016). Rare intronic and splice site variants were assessed using SpliceAI (Jaganathan et al., 2019). Fetal sex was inferred by non-pseudoautosomal region X-chromosome genotype analysis using the bcftools guess-ploidy plugin, allowing for genotyping error rate of 0.1.

A careful delineation of the URSMS subtypes and anomalies in other systems was carried out. Literature was reviewed for syndromes or the phenotypes associated with URSMS. We searched PubMed, Online Mendelian Inheritance in Man and other databases with the phrases “urorectal septum malformation sequence,” “persistent cloaca,” “cloacal dysgenesis,” and “cloaca” to look for the associated malformations.

## 3 Results

We observed URSMS in 12 fetuses (5%) in our cohort of 215 fetal losses. The gestational age ranged 13–36 weeks. Ambiguous genitalia with no perineal openings and persistent cloaca characterizing the complete URSMS was observed in nine (9/12, 75%) fetuses. There was persistent cloaca with a single perineal opening (partial URSMS) in two fetuses and the other fetus had intermediate type (complete partition of the persistent cloaca without perineal openings) of URSMS. Oligohydramnios (4/12, 33%) or renal agenesis/anomaly (4/12, 33%) or megacystis (1/12, 8%) were observed prenatally in the cohort. The clinical details of all the fetuses are provided in Table 1. Overall, we could identify sex in five fetuses based on external and internal examination and histopathology. Recurrence of the condition was not observed in any of the families in our cohort.

Eleven (11/12, 91.6%) fetuses had malformations (Table 1) outside the urinary tract, internal and external genital and hindgut. Anomalies of vertebrae (10/12, 83%), limbs (8/12, 66%), central nervous system (5/12, 41%), umbilical cord (4/11, 36%), heart (3/12, 25%), and ventral body wall (3/12, 25%) were frequently noted in our cohort.

### 3.1 Unique malformations with URSMS

After reviewing the literature for syndromes or conditions with multiple malformations where URSMS is a component, we note, five fetuses had a rare or unique pattern of multiple malformations in our cohort. These rare presentations are described here.

#### 3.1.1 Complete URSMS with cerebral ventriculomegaly (Fetus 2)

**Figure 1:**
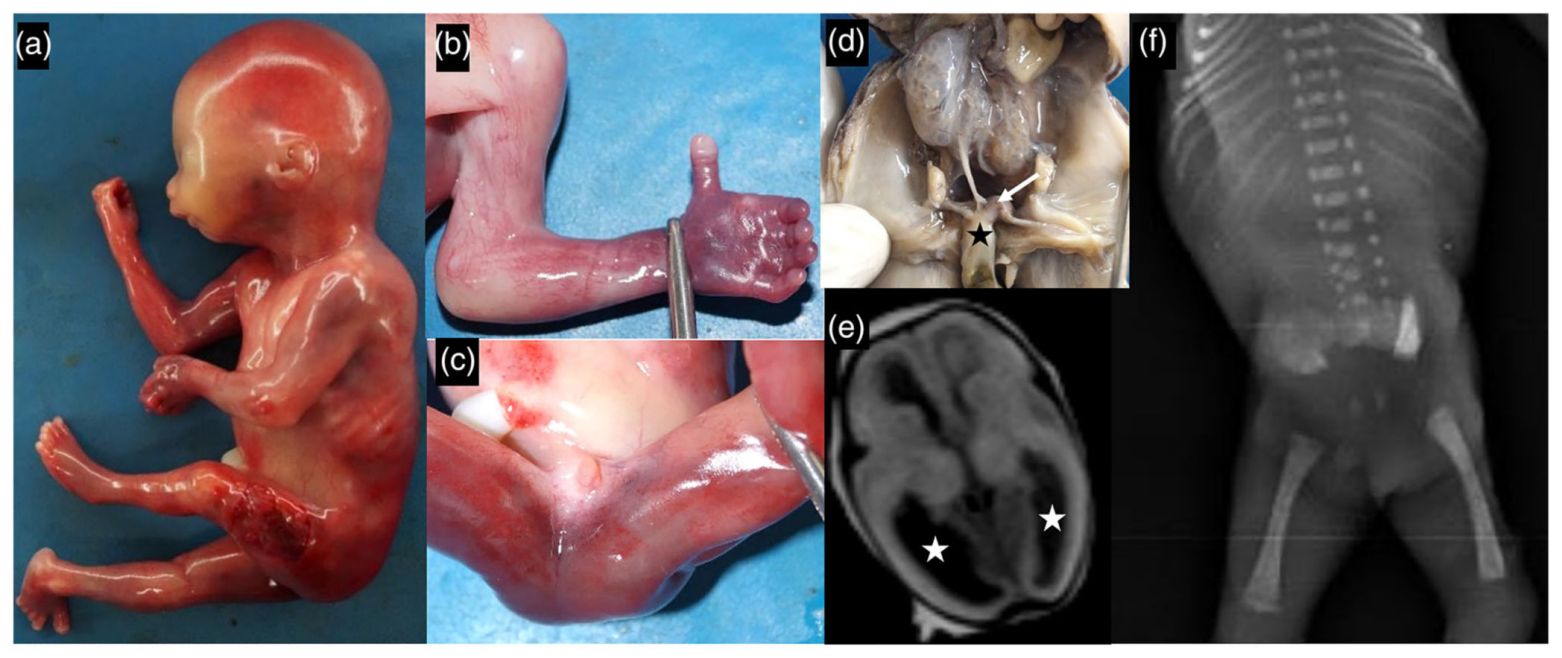
Depressed nasal bridge, micro-retrognathia, contractures in upper and lower limbs (a), proximally placed left thumb (b), ambiguous external genitalia with absent perineal openings (c) and multicystic and fused kidneys with narrow ureters and hindgut (black asterisk, d) draining into common, blind cloaca (arrow, d) were observed in Fetus 2. Imaging revealed cerebral ventriculomegaly (white asterisk, e) and absence of ossification of sacral vertebrae (f).

The fetus was ascertained at 18 weeks of gestation in view of oligohydramnios and bilateral renal agenesis. The fetus had normal anthropometric measurements (weighed 175 g, measured 22 cm in length with head circumference of 15 cm). Depressed nasal bridge, micro-retrognathia, proximally placed left thumb, contractures across elbows, hips and knees, calcaniovalgus of right foot and two vessels umbilical cord were seen. Ambiguous external genitalia with a small phallus like structure and absence of perineal openings was noted. Dilated lateral cerebral ventricles were evident on gross examination and magnetic resonance imaging of fetal brain (Figure 1). Kidneys were multicystic and fused, with narrow ureters draining into blindending cloaca. Rudimentary allantois and indifferent gonads were observed. Radiographs of the fetus showed the absence of ossification of sacral vertebrae.

#### 3.1.2 Intermediate URSMS, right aortic arch with double outlet right ventricle (Fetus 3)

**Figure 2:**
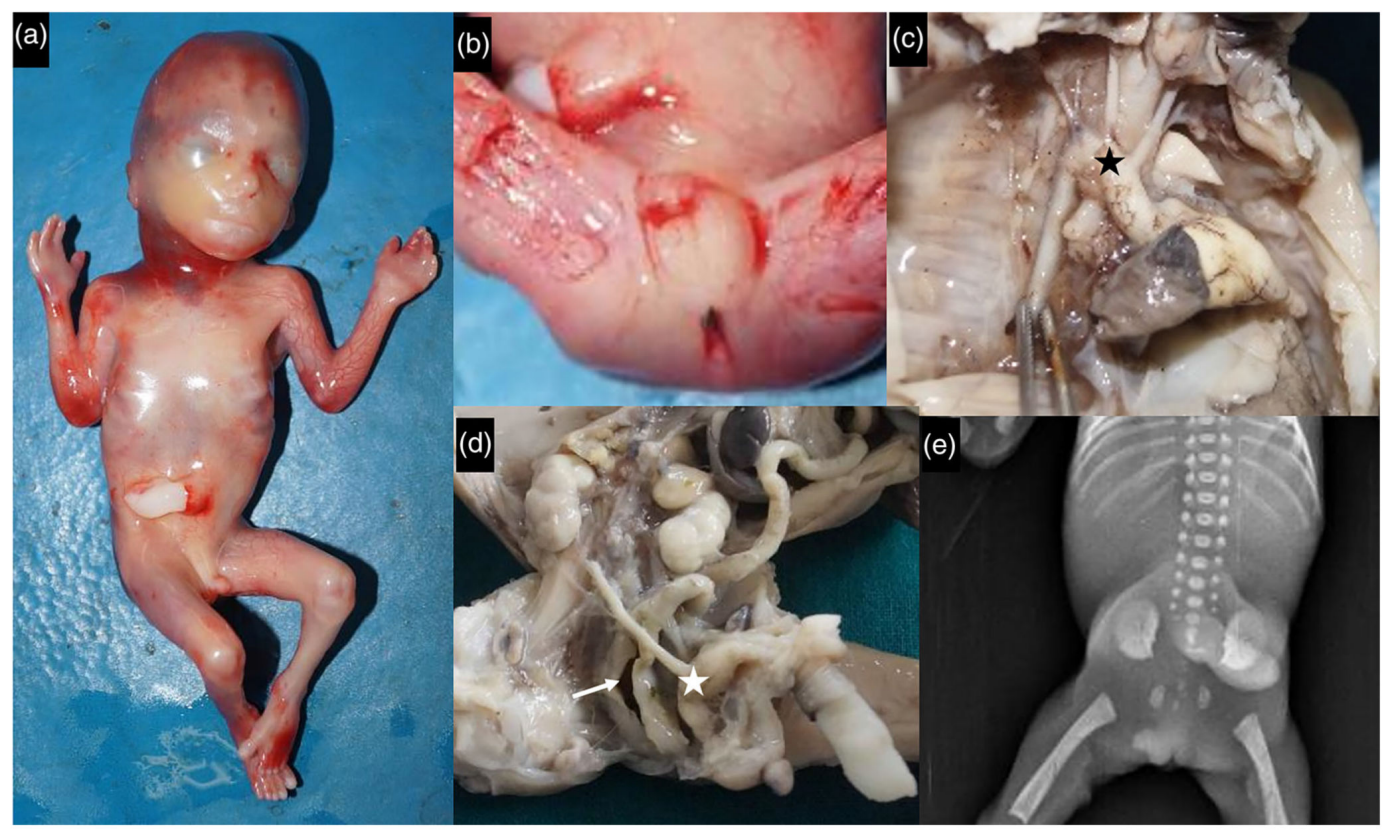
Fetus 3 had short nose, long philtrum, retrognathia (a), small phallus with scrotum and imperforate anus (b), right aortic arch (black asterisk, c), hypoplastic kidneys, separate rectum (arrow) and urinary bladder (white asterisk) with no perineal openings (d) and poorly ossified sacral vertebrae (e).

A third gravida with diabetes mellitus and hypothyroidism was noted to have anhydramnios, symmetric early onset of growth retardation and cardiac defect in the fetus antenatally. Evaluation of fetus showed normal anthropometry (corresponding to 18 weeks of gestation) with short nose, long philtrum, retrognathia, imperforate anus, small phallus with small scrotum, and single umbilical artery. Right aortic arch with double outlet right ventricle, sub-aortic ventricular septal defect, hypoplastic left ventricle, and nonlobulated lungs were evident (Figure 2). Both kidneys were hypoplastic and ureters draining into the urinary bladder. The urethral opening was absent and gonads appeared to be testes. There was absence of ossification centers for sacrum.

#### 3.1.3 Partial URSMS, microcephaly, and fetal akinesia deformation sequence (Fetus 4)

**Figure 3:**
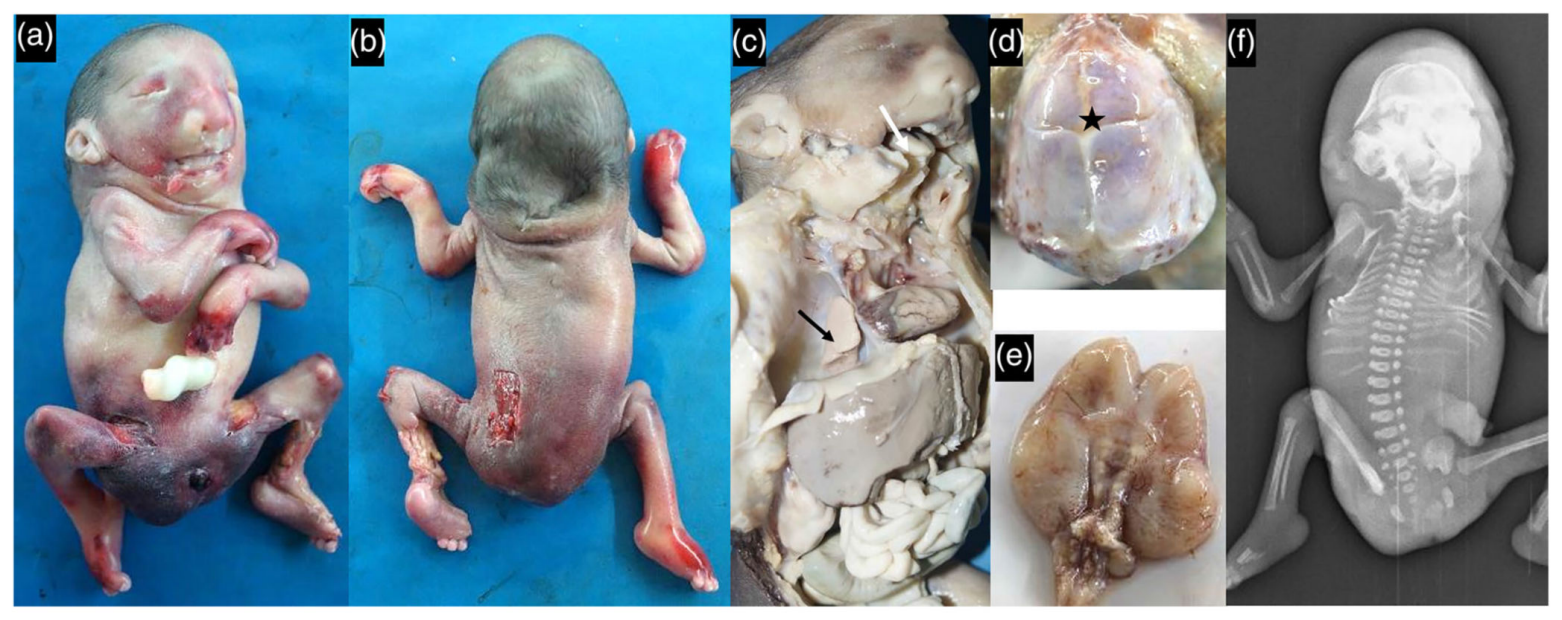
Microcephaly, sloping forehead, dysmorphic ears, hypertelorism, midface protrusion, macrostomia and micro-retrognathia, short neck with webbing, pterygia across axillae, elbows, hips and knee joints, ambiguous external genitalia (a, b), ankyloglossia (white arrow), hypoplastic lungs (black arrow, c), premature closure of fontanelle (asterisk, d), small brain (e) and scoliosis, crowding of ribs and hyper-mineralized cranium (f) were evident in Fetus 4.

The fetus weighed 256 g (–3.1 SD) measured 17 cm (–6.8 SD) in length with head circumference of 15 cm (–4.5 SD) at 24 weeks of gestation. Trigonocephaly was noted with fused anterior fontanel and excessive skin on the occipital and neck region, sloping forehead, dysmorphic ears, hypertelorism, midface protrusion, wide mouth, and micro-retrognathia. The fetus also had short neck with webbing, pterygia across the axillae, elbows, hips and knees, camptodactyly, over-riding of fingers, bilateral congenital talipes equinovarus, ambiguous external genitalia with common urogenital opening and atresia of anal canal (Figure 3).

Further examination disclosed underdeveloped brain, ankyloglossia, severely hypoplastic lungs, absence of muscle mass in upper and lower limbs, atresia of terminal portion of hindgut, presence of ovaries and uterus, vesicovaginal fistula with common perineal opening. Scoliosis, crowding of ribs, hyper mineralized cranium, and small head were noted on radiography. Fetal karyotype and chromosomal microarray were normal. Exome sequencing from fetal DNA was nondiagnostic.

#### 3.1.4 Partial URSMS with amelia (Fetus 11)

**Figure 4:**
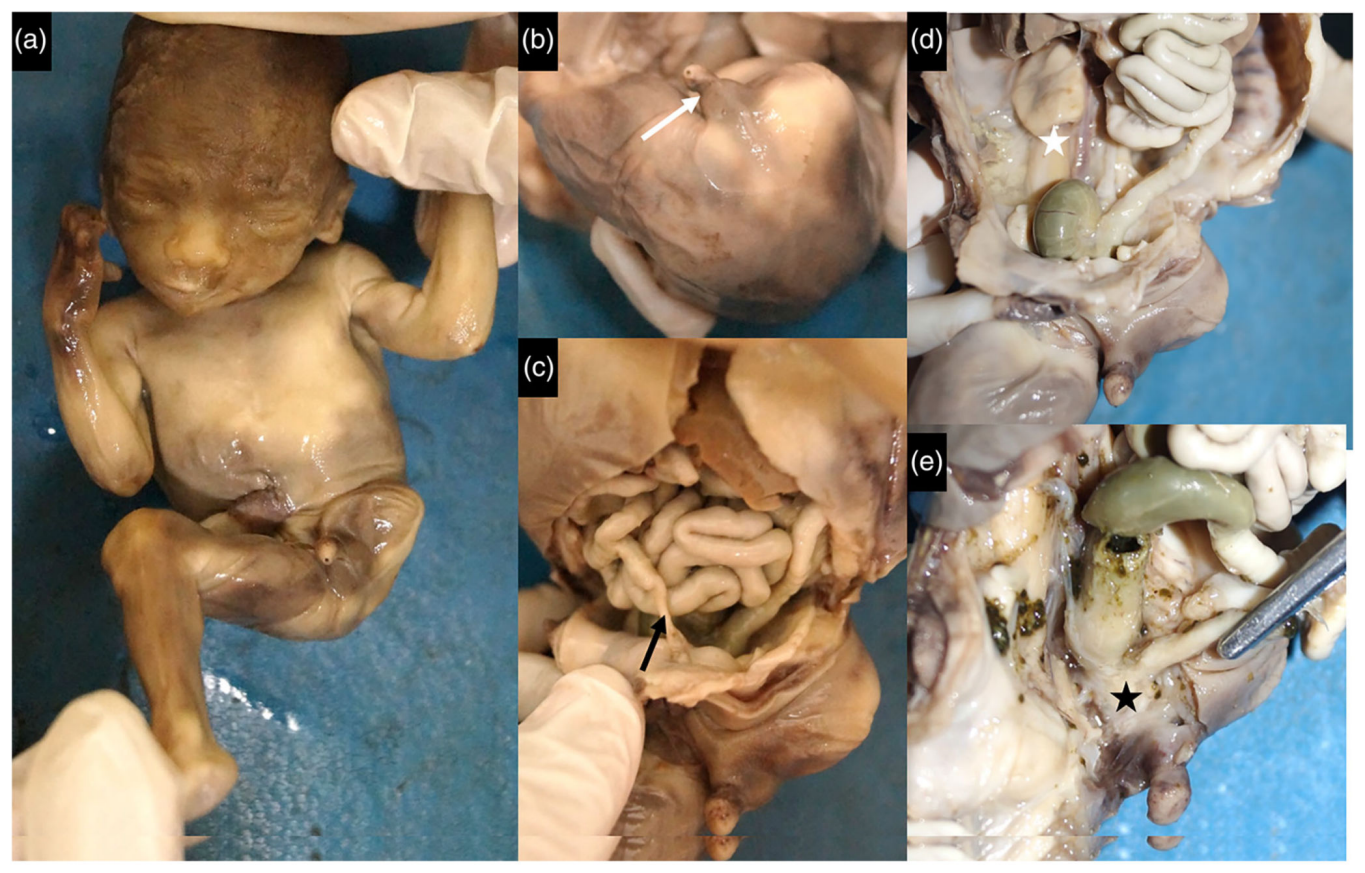
There was amelia of left lower limb (a), phallus-like structure with single perineal opening (white arrow, b), Meckel’s diverticulum (black arrow, c), bilateral renal agenesis (white asterisk, d), incomplete separation of rectum, and rudimentary bladder with common outlet (black asterisk, e) in Fetus 11.

Prenatal diagnosis disclosed bilateral renal agenesis with absent urinary bladder, malformed left lower limb and intrauterine growth retardation in a fetus. The fetus weighed 223 g (–0.3 SD) measured 19 cm (–2 SD) in length at 19 weeks of gestation. Hypertelorism, retrognathia, proximally placed right thumb, and complete absence of left lower limb were evident. There was a phallus-like structure with a single perineal opening. Persistent vitellointestinal duct (Meckel's diverticulum), bilateral renal agenesis, and absent ureters with rudimentary urinary bladder were noted in the fetus (Figure 4). The rectum was communicating with the lower part of bladder and was drained by a common perineal opening. Gonads were confirmed to be testes on histopathology.

#### 3.1.5 URSMS, abdominal wall defect, limb contractures with cleft foot (Fetus 12)

A primigravida with history of laminectomy for scoliosis, polycystic ovarian disease and hypothyroidism underwent pregnancy interruption at 12 weeks of gestation. Anterior abdominal wall defect with herniation of liver, cystic area in lower portion of abdomen, and posterior bending of lower limbs were observed on ultrasonography.

**Figure 5:**
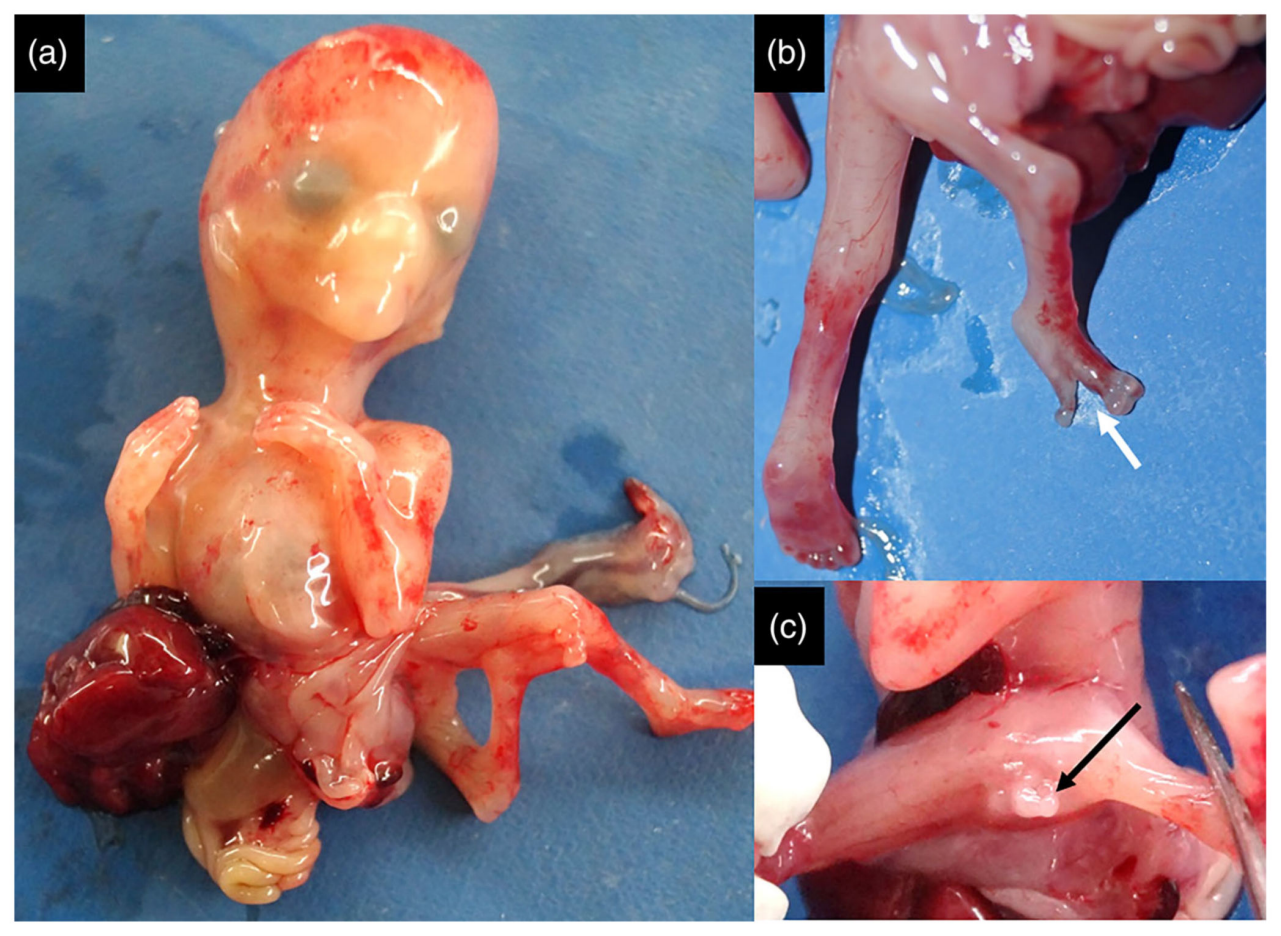
Images depict anterior abdominal wall defect with herniation of abdominal viscera, kyphoscoliosis (a), contractures and spilt right foot (white arrow, b) and ambiguous external genitalia with absent perineal openings (black arrow, c) in Fetus 12.

Growth parameters of the fetus were within normal limits. There was an anterior abdominal wall defect with herniation of liver, stomach, spleen, pancreas, and loops of small and large intestine partially covered by peritoneum. Severe kyphoscoliosis at the thoracolumbar region, short right lower limb with joint contractures across hip, knee, and ankle were noted (Figure 5). Right cleft foot was observed with absence of two central digits. Imperforate anus, ambiguous external genitalia, indifferent gonads, a defect in diaphragm, agenesis of right kidney and right adrenal gland, persistent cloaca with hindgut and left ureter draining into it, and atresia of cloacal opening were additional findings.

### 3.2 Recurrent malformations with URSMS

We observed several recurrent malformations that are already known to occur with URSMS in six fetuses of our cohort. They include radial ray defect, unilateral renal agenesis, and ventricular septal defect suggestive of vertebral defects, anal atresia, tracheoesophageal fistula with esophageal atresia and radial or renal dysplasia (VATER) (Fetus 5); thoraco-abdominoschisis and limb defects (Fetus 6); myelomeningocele/occipital encephalocele, renal agenesis with vertebral segmentation defect (Fetuses 7 and 10); spina bifida and fused iliac bones (Fetus 8) and omphalocele and scoliosis (Fetus 9). The antenatal history, pedigree, clinical findings, and images of the fetuses with recurrent phenotypes are provided in supplementary information S1.

### 3.3 Genomic testing

Genomic testing was performed in eight (8/12) families. Fetal karyotype was available for Fetus 3 and Fetus 4 following prenatal invasive testing and showed normal chromosomes. In Fetus 4, chromosomal microarray did not reveal any clinically significant copy number variations. Singleton exome sequencing (nondiagnostic) was carried in Fetus 4. Fetuses 1, 3, 5, 8, and 9 underwent singleton genome sequencing, whereas Fetuses 2, 4, and 6 underwent trio-genome sequencing. Genetic analysis inferred Fetuses 1, 4, 5, and 9 were female, and Fetuses 2, 3, 6, and 8 were male. Data analysis did not identify any pathogenic single nucleotide variants in known genes associated with the URSMS phenotype, but variants of interest have been identified in Fetuses 1, 3, 8, and 9 (Table 2).

## 4 Discussion

In this study, we report on 12 (5%) fetuses with URSMS and describe the associated pattern of malformations in them. We note complete URSMS in the majority of the fetuses (9/12, 75%), partial URSMS in two fetuses, and intermediate URSMS in one fetus. Eleven fetuses (91.6%) had multiple malformations affecting other systems and one had isolated URSMS. Overall, six fetuses had recurrent and five fetuses had unique malformations in our cohort. We identified variants of interest in four fetuses (Table 2).

There have been reports in the literature on the clinical presentation of fetuses with URSMS (Jain et al., 2008; Patil & Phadke, 2006; Tennant et al., 2014; Wheeler et al., 1997; Wheeler & Weaver, 2001) and a previous cohort from our center (Shah et al., 2016). The rate of associated anomalies of other systems ranges from 65% to 89% (Jain et al., 2008; Patil & Phadke, 2006; Tennant et al., 2014; Wheeler et al., 1997; Wheeler & Weaver, 2001). Limb malformations (e.g., club foot, polydactyly, limb reduction defects, absent/hypoplastic thumb, absent fibula, hip dislocation, cubitus valgus, genu valgum, clinodactyly, and simian creases) and cardiac anomalies (atrial septal defect, ventricular septal defect, tetralogy of Fallot, tricuspid stenosis, pulmonary valve stenosis, truncus arteriosus, atrioventricular canal defect, anomalous venous return, and patent ductus arteriosus) appear to be the most frequent (in 32% each) followed by vertebral anomalies, mainly affecting the lumbar spine in 22.8%; central nervous system anomalies (neural tube defects and holoprosencephaly) in 16.5%; gastrointestinal anomalies (tracheoesophageal fistula, esophageal atresia, small gut atresia or stenosis, Meckel's diverticulum and atresia of bile duct) in 15.7%; ventral body wall defect/omphalocele in 8.6%; single umbilical artery/urachal cyst in 7% and others (congenital diaphragmatic hernia, congenital cystic adenomatoid malformation of lung, cleft palate, perineal hernia) very rarely.

The recurrent malformations or phenotypes observed in our cohort were VATER association, limb body wall defect, neural tube defects and abdominal wall defect. Neural tube defects were more common in our small cohort. Prune-belly sequence and vertebralanal-cardiac-tracheoesophageal fistula-renal-limb (VACTERL) association were also observed with URSMS earlier (Tennant et al., 2014). Patterns of severe ventral body wall defects were studied by Vauthay et al. and they report cloacal anomalies in 76.9% (Vauthay et al., 2007). Heyroth-Griffis et al. (2007) proposed that exstrophy of cloaca, URSMS and limb body wall complex (LMBW) represented a continuous spectrum of anomalies in view of overlapping features (Heyroth-Griffis et al., 2007). LMBW, Mullerian duct aplasia-renal anomalies-cervicothoracic somite dysplasia (MURCS), oculoauriculovertebral spectrum (OAVS), omphalocele-exstrophy-imperforate anus-spinal defects (OEIS) complex, pentalogy of Cantrell (POC) and VATER/VACTERL are developmental disorders occurs during early embryogenesis (Adam et al., 2020). They are termed as recurrent constellation of embryonic malformations (RCEM) and constitute a spectrum and are likely to be causally related. We hypothesize that the URSMS also likely belongs to RCEM based on the overlapping features with LMBW, MURCS, and VACTERL. The highlight of our work is documentation of some of the rare malformations associated with URSMS such as cerebral ventriculomegaly, right aortic arch with double outlet right ventricle, fetal akinesia deformation sequence, amelia and limb contractures with spilt foot that are not described earlier, to the best of our knowledge.

Many dysmorphic features and contractures could be caused by oligohydramnios. Antenatally oligohydramnios or anhydramnios was noted in four fetuses (Fetuses 2, 3, 7, and 10) in our cohort. Fetus 2 had contractures of all major limb joints; Fetus 10 had contractures of limb joint along with short thigh, whereas facial dysmorphism was observed in Fetuses 3 and 10, which may be secondary to oligohydramnios.

One of the twins from a monochorionic diamniotic pregnancy (Fetus 9) had URSMS and associated malformations. Twinning with URSMS was noted previously in the literature (Achiron et al., 2000). The familial occurrence of URSMS is very rare and has been reported in two families (Aggarwal & Phadke, 2013; Mills & Pergament, 1997). One of them had a dominant inheritance, where the mother had mild manifestations and the child had severe URSMS (Mills & Pergament, 1997), whereas the other family had prune belly in the first sib and URSMS in the second sib suggesting a recessive pattern of inheritance (Aggarwal & Phadke, 2013). These observations suggest the existence of a (mono) genetic etiology, at least for a proportion of patients with URSMS.

The cloaca is an embryonic structure evident by the fourth week of development and is divided to form urogenital sinus and primitive rectum by sixth week of intrauterine life. Hence, we speculate the genes involved in the early development of cloaca—Sonic hedgehog (Shh), SOX2 and CDX2, which express in the cloacal epithelium; Six1 and Ey1 intrinsic regulator of mesenchyme surrounding cloaca; Gli2 and Gli3 downstream mediator of Shh and BMP signaling are likely to play a role in pathophysiology (Runck et al., 2014).

The *TMEM132A* missense variant identified in fetus 1 may be relevant to the persistent cloaca observed in this fetus. *Tmem132a*^−/−^ mice have impaired hind limb growth and spina bifida in addition to bladder and kidney defects (Li et al., 2022). Whole embryo staining of *Tmem132a*^−/−^ mice revealed *Cdx2* is notably downregulated in the hindgut (Li et al., 2022). The importance of CDX2 signaling in the normal development of the cloacal epithelium is supported by the identification of damaging de novo variants in CDX2 in two unrelated patients with persistent cloaca (Hsu et al., 2018). Recently, variants in *CDX2* have also been reported in two familial aggregations with phenotype ranging from sirenomelia with different degrees of urogenital malformations to isolated imperforate anus indicating its role in caudal malformations (Lecoquierre et al., 2020). Moreover, the identification of a missense variant in *HOXD9* in Fetus 8 is also relevant to the *Shh/CDX2* pathway. Reduction in *Hoxd9* expression observed in embryonic limb buds of rats supplemented with all-trans-retinoic acid correlated with impaired *Shh/Gli3* signaling and reduced *Sox9/Col2a11* signaling (Hong et al., 2021). Both *Shh* (Seifert et al., 2009) and *Gli3* (Mo et al., 2001) expression are reduced in rats with anorectal malformations (Liu et al., 2016). Additionally, a disorganization-like syndrome was noted with persistent cloaca, having an incomplete duplication of left lower limb (Al Kaissi et al., 2008), suggesting an overlap in signal defects can cause deficiencies in both tissues. The normal limb development observed in Fetus 8 suggests the *HOXD9* c.277A > G variant would only urorectal development would be affected in this case, despite the association of *HOXD9* with limb defects.

Moreover, The *SLIT2* variant identified in Fetus 8 may relate to the unilateral renal agenesis observed during fetal evaluation. *Slit2*^−/−^ mice develop multiple ureters and fused dysplastic kidneys as a result of ureteric buds, which remain inappropriately connected to the nephric duct during development (Grieshammer et al., 2004). Furthermore, heterozygous missense *SLIT2* variants were identified in three unrelated patients from a cohort with congenital anomalies of the kidney and urinary tract (CAKUT), one patient with bilateral subcortical renal cysts, one patient with right multicystic dysplastic kidneys, and one patient with right renal agenesis (Hwang et al., 2015).

In Fetus 8, the identification of concomitant *HOXD9* and *SLIT2* missense variants prompts the hypothesis that the etiology of URSMS in this fetus is oligogenic. URSMS has been identified as a component in a few syndromes/phenotypes, which are listed in Online Mendelian Inheritance in Man along with their genetic bases: persistent cloaca and prune belly syndrome with tricho-rhino-phalangeal syndrome type II (MIM#150230) associated with 8q interstitial deletion (Ramos et al., 1992); Fanconi anemia, complementation group A (MIM# 227650) (Farrell et al., 1994); sacral agenesis with vertebral anomalies (MIM# 615709) due to biallelic variants in *TBXT* (Postma et al., 2014); Omphalocele-Exstrophy-Imperforate Anus-Spinal Defects (OEIS complex, MIM 258040) associated with 1p36 deletion (El-Hattab et al., 2010) and a heterozygous variants in Uroplakin 3A associated with renal dysplasia and persistent cloaca (Jenkins et al., 2005). The London Medical Database has following entities listed with exstrophy of cloaca: caudal duplication syndrome; caudal regression; diphallus plus; femoral duplication; frontonasal dysplasia-exstrophy of bladder or cloaca; Gollop-Wolfgang complex caused by *BHLHA9*; sirenomelia; urorectal septal defects and VATER association.

Several researchers explored the genetic basis of URSMS and related anomalies. Patients with anorectal malformations and VACTERL association or cloacal exstrophy were analyzed for variants in *FGF10* following a reproducible phenotype of urorectal defect in mice; however, the data obtained were not supportive (Krüger et al., 2008). Isochromosome 18q was observed in a fetus with congenital megacystis, cloacal dysgenesis sequence and median cleft lip and palate by karyotype (Chen et al., 1998). Array comparative genomic hybridization in 17 females with cloaca identified novel copy number variations (paternally inherited duplication on 16p13.2 and a de novo deletion on 1q32.1q32.3) in two patients (Harrison et al., 2014). The latter deletion included a plausible candidate, hedgehog acyltransferase (*HHAT*); however, Sanger sequencing of independent patients with cloacal defects did not identify any significant variants in this gene. Prenatal diagnosis by chromosomal microarray in a pregnancy suspected with URSMS detected paternally inherited 111.8Kb deletion at 16p13.3 in the affected fetus (Pei et al., 2016). Similarly, *ZNF157, SP8, ACOT9*, and *TTLL11* are thought to be associated with VATER/VACTERL following exome sequencing in a cohort of 21 families (Kolvenbach et al., 2021). However, further studies are required to explain the causality.

We identified heterozygous missense *NALCN* variants in Fetuses 3 and 9. *NALCN* encodes the nonselective sodium leak channel, and homozygous knockout is incompatible with life in mice (Cochet-Bissuel et al., 2014). Previously, de novo *NALCN* variants were observed in 14 unrelated probands with congenital contractures of the limbs and face, hypotonia and developmental delay (CLIFAHDD, MIM#616266) (Chong et al., 2015). *NALCN* is primarily expressed in the nervous system, suggesting its role is in movement coordination and intellectual development, rather than tissue patterning and development (Kasap et al., 2017). Phenotypes in Fetuses 3 and 9 are do not feature contractures, while hypotonia and developmental delay were not discernible in this cohort. However, scoliosis, clubfoot, and omphalocele observed in Fetus 9 are reminiscent of the additional phenotypes observed among the CLIFAHDD cohort (Chong et al., 2015).

We assume that the presence of various malformations with URSMS represent clinically diverse spectrum of perinatal lethal syndromes with URSMS. Our observations call for a concerted effort for delineation of clinically and likely etiologically heterogeneous conditions with URSMS. Genome sequencing and analysis did not identify variants in known URSMS genes, but it identifies novel candidate variants that provide the basis for future investigations. However, in some cases, no variant of interest was identified, which suggests that these fetuses may have a more complex oligo/polygenic etiology with environmental influences.

## Supplementary Material

Supplementary file

## Figures and Tables

**Table 1:** Clinical features in fetuses with urorectal septum malformation sequence (URSMS)

Features	Fetus 1	Fetus 2	Fetus 3	Fetus 4	Fetus 5	Fetus 6	Fetus 7	Fetus 8	Fetus 9	Fetus 10	Fetus 11	Fetus 12
Gestational age in weeks	13	18	18	24	20	14	36	Not available	14	30	21	12
Gender	Female	Unknown	Male	Female	Female	Unknown	Unknown	Unknown	Unknown	Unknown	Male	Unknown
Consanguinity	No	No	No	No	No	No	No	Not available	No	No	No	No
Gravida	Primi	Second	Third	Primi	Second	Second	Second	Not available	Primi	Second	Primi	Primi
Antenatal events	Fever in first trimester	Nil	Diabetes mellitus and hypothyroidism	Nil	Nil	Nil	Nil	Not available	Twin pregnancy	Nil	Nil	Hypothyroidism and polycystic ovarian disease
Type of URSMS	Complete	Complete	Intermediate	Partial	Complete	Complete	Complete	Complete	Complete	Complete	Partial	Complete
External genitalia	Small phallus-like structure with no urogenital openings	Small phallus-like structure with no urogenital openings	Small phallus-like structure with scrotum with no urethral opening	Ambiguous genitalia with no urogenital openings	Small phallus-like structure with no urogenital openings	Small phallus-like structure with no urogenital openings	Small phallus-like structure with no urogenital openings	Small phallus-like structure with no urogenital openings	Small phallus-like structure with no urogenital openings	Ambiguous genitalia with no urogenital openings	Phallus-like structure with a perineal opening	Small phallus-like structure with no urogenital openings
Anal opening	Absent	Absent	Absent	Present	Absent	Absent	Absent	Absent	Absent	Absent	Absent	Absent
Persistent cloaca	Present (blind ending)	Present (blind ending)	Urinary bladder with urethral opening	Vesico-vaginal fistula with common opening	Present (blind ending)	Present (blind ending)	Present (blind ending)	Present (blind ending)	Present (blind ending)	Present (blind ending)	Recto-vesicle fistula with common perineal opening	Present (blind ending)
Internal genitalia	Ovaries, fallopian tube	Indifferent gonads	Testes	Ovaries and uterus	? Ovaries, no uterus	Indifferent gonads	Gonads were not found	Indifferent gonads	Indifferent gonads	Indifferent gonads	Testes	Indifferent gonads
Hindgut	Draining into cloaca	Draining into cloaca	Blind ending	Atresia	Draining into cloaca	Draining into cloaca	Draining into cloaca	Draining into cloaca	Draining into cloaca	Draining into cloaca	Draining through common perineal opening	Draining into cloaca
Renal agenesis (unilateral/ bilateral)	No	No	No	No	Left renal agenesis	Left renal agenesis (with adrenal)	Bilateral renal agenesis	Left renal agenesis	No	Bilateral renal agenesis	Bilateral renal agenesis	Right renal agenesis
Cystic kidneys	Present (horseshoe)	Present (fused)	Hypoplastic	Absent	Absent	Absent	Absent	Absent	Absent	Absent	Absent	Absent
Umbilical cord	Three vessel	Two vessel	Two vessel	Three vessel	Two vessel	Not available	Three vessel	Three vessel	Three vessel	Two vessel	Three vessel	Three vessel
Sacrum	Absent ossification	Absent ossification	Reduced ossification	Normal	Normal	Absent ossification	Absent ossification	Absent ossification	Absent ossification	Absent ossification	Normal	Absent ossification
Central nervous system anomalies	Absent	Dilated lateral cerebral ventricles	Absent	Small brain	Absent	Absent	Lipomeningomyelocele	Lumbar spina bifida	Absent	Occipital encephalocele	Absent	Absent
Cardiac anomalies	Absent	Absent	Right aortic arch, double outlet right ventricle with ventricular septal defect	Absent	Membranous ventricular septal defect	Dextrocardia	Absent	Absent	Absent	Absent	Absent	Absent
Limb anomalies	Absent	Proximally placed left thumb, contractures across elbows, hips and knees, talipes equinovalgus of right foot	Absent	Bilateral congenital talipes equinovarus	Bilateral radial deviation of hands, bowed fore arms, rudimentary right thumb with absent radius, absent F1 and F2 on left hand with hypoplastic radius	Short right lower limb with contractures, reduction defect of left lower limb below the knee with bowed femora	Right radial club hand, bilateral clubfeet and rhizomelia of left lower limb	Absent	Unilateral club foot	Absent	Proximally placed right thumb, complete absence of left lower limb	Short right lower limb with joint contractures across hip, knee and ankle, right cleft foot with absent two central digits (split foot)
Skeletal abnormalities	Absent	Absent	Absent	Scoliosis, crowding of ribs and hyper mineralization cranium	Absent	Kyphoscoliosis at thoracolumbar region	Lumbar scoliosis, segmentation defect of thoracic and lumbar vertebrae	Fused iliac bones, absence of ossification centers for lower lumbar vertebrae	Scoliosis at lumbar region	Segmentation defect in lower lumbar vertebrae	Absent	Kyphoscoliosis at thoracolumbar region
Ventral body wall defect	Absent	Absent	Absent	Absent	Absent	Lower thoracic and abdominal wall defect with the herniation	Absent	Absent	Omphalocele	Absent	Absent	Anterior abdominal wall defect with the herniation
Other anomalies	Protuberant abdomen with thin and transparent abdominal wall	Rudimentary allantois	Nonlobulated lungs	Microcephaly, excessive skin on the occipital and neck region, sloping forehead, dysmorphic ears, hypertelorism, midface protrusion, macrostomia, micro-retrognathia, short neck, pterygia, camptodactyly, overriding fingers, craniosynostosis, ankyloglossia, hypoplastic lungs	Facial asymmetry on right side, hypertelorism, short nose, ante verted nares, long philtrum and retrognathia	Absent	Pulmonary hypoplasia	Absent	Absent	Frontal sloping, flattened nasal tip, micrognathia, dysmorphic ears, short neck, bilateral pulmonary hypoplasia	Hypertelorism, retrognathia, persistent vitellointestinal duct	Absent
Fetal karyotype	Not done	Not done	Done (Normal)	46, XX	Not done	Not done	Not done	Not done	Not done	Not done	Not done	Not done
Chromosomal microarray	Not done	Not done	Not done	Normal	Not done	Not done	Not done	Not done	Not done	Not done	Not done	Not done
Genome sequencing	Singleton	Trio	Singleton	Trio (Also underwent singleton exome)	Singleton	Trio	Not done	Singleton	Singleton	Not done	Not done	Not done
Inferred genomic sex	Female	Male	Male	Female	Female	Male	Not done	Male	Female	Not done	Not done	Not done

**Table 2:** Variants of interest identified from WGS of eight fetuses with URSMS

Fetus	Gene	Zygosity	GRCh37 coordinate	cDNA	Protein	GnormAD missense z-score (observed/expected)	GnormAD AF	GnormAD south Asian AF	Align GVGD	Mutation taster	Polyphen2	SIFT	CADD	REVEL	ACMG classification	ACMG evidence
1	*TMEM132A*	Het	11:60701998A > G	NM_017870.2: c.1601A > G	p.Glu534Gly	0.8 (590/647)	0	0	C0	Benign	Possibly Damaging (0.787)	Tolerated (0.27)	23.6	0.13	Uncertain Significance	PM2, PB4
3	*NALCN*	Het	13:101712309 T > A	NM_052867.4: c.4766A > T	p.Gln1589Leu	4.96 (563/1005.1)	0	0	C0	Deleterious	Possibly Damaging (0.93)	Deleterious (0.01)	23.3	0.864	Uncertain Significance	PM2, PP2, PP3
8	*HOXD9*	Het	2:176987773A > G	NM_014213.4: c.277A > G	p.Ser93Gly	0.58 (147/168)	0	0	C0	Benign	Possibly Damaging (0.789)	Tolerated (0.10)	22.9	0.383	Uncertain Significance	PM1, PM2, BP4
8	*SLIT2*	Het	4:20620441G > A	NM_004787.4: c.4399G > A	p.Gly1467Ser	2.1 (687/860)	0	0	C55	Deleterious	Probably Damaging (1.00)	Deleterious (0.00)	29.6	0.51	Uncertain Significance	PM1, PM2, PP3
9	*NALCN*	Het	13:102051423C > A	NM_052867.4: c.55G > T	p.Gly19Cys	4.96 (563/1005)	0	0	C0	Deleterious	Probably Damaging (0.999)	Deleterious (0.01)	27.7	0.805	Uncertain Significance	PM2, PP3

Abbreviations: AF, allele frequency; URSMS, urorectal septum malformation sequence, WGS, whole genome sequencing.

## Data Availability

The data that support the findings of this study are available in the supplementary material of this article.
