# Supplementary material for: Clinically diverse and perinatally lethal syndromes with urorectal septum malformation sequence
Source: Am J Med Genet A. Author manuscript; Available in PMC 2024 Apr 24. (PMC7615864; doi:10.1002/ajmg.a.63067)
Supplement: Supplementary file [file EMS195514-supplement-Supplementary_file.docx]

**A clinically diverse spectrum of perinatal lethal phenotypes with urorectal septum malformation sequence**

**Supporting information**

**Fetus 1 (Complete urorectal septum malformation sequence)**

**Pedigree:**


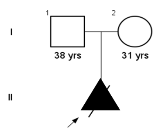


13 weeks


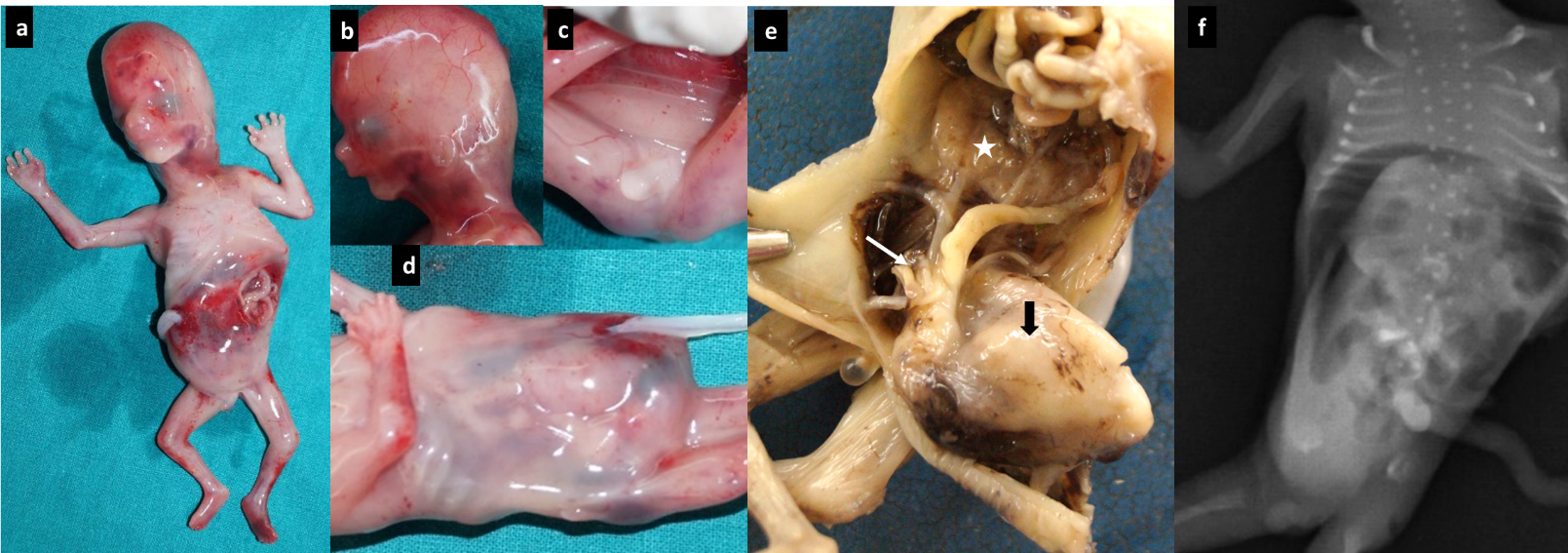


**Supplementary figure 1**: Clinical images of fetus 1 show short nose with depressed nasal root, anteverted nares, long philtrum and retrognathia (a, b), protuberant abdomen with thin and transparent wall (a, d), ambiguous external genitalia with imperforate anus (c), multi-cystic and fused kidneys (white asterix) with narrow ureters, fallopian tube (white arrow) and hind gut draining into blind cloaca (black arrow, e) and absent ossification of sacrum (f).

**Clinical findings:** The fetus was examined at 13 weeks of gestation in view of megacystis on antenatal ultrasonography. Fetus weighed 31 g (normal), measured 12 cm in length (normal) with head circumference of 8.5 cm (normal). Fetus had short nose with depressed nasal root, anteverted nares, long philtrum and retrognathia. Protuberant abdomen was observed with thin and transparent abdominal wall. Ambiguous external genitalia with a small phallus-like structure with no perineal openings were noted. Multi-cystic and fused kidneys were observed with ureters running anterior to the fused part of kidney. The ureters, fallopian tubes and hind gut were connected to the enlarged and persistent blind cloaca (Supplementary figure 1). Radiographs of the fetus showed absence of ossification centers for sacrum.

**Fetus 5 (Radial ray defect, unilateral renal agenesis, urorectal septum malformation sequence and ventricular septal defect)**

**Pedigree:**


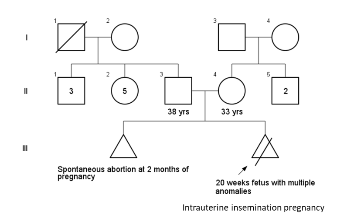


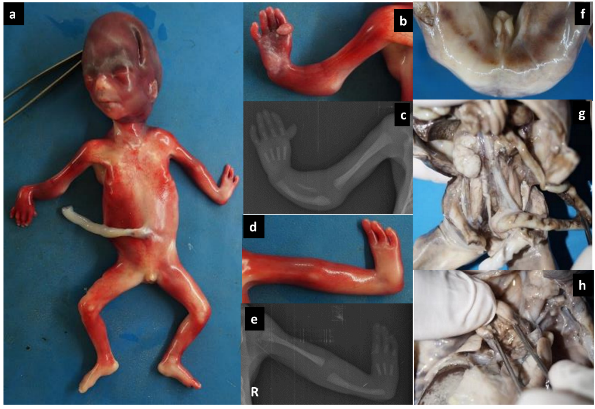
 **Supplementary figure 2**: We observed facial asymmetry with hypertelorism, short nose, anteverted nares, long philtrum, retrognathia (a), right upper limb with small radius and rudimentary thumb (b, c), left upper limb with complete absence ray 1 and 2 with absent radius (d, e), ambiguous genitalia (f), unilateral renal agenesis with blind ending cloaca (asterisk, g) and ventricular septal defect (arrow, h) in fetus 5.

**Clinical features:** Abortion in a second gravida at 20 weeks of gestation with hypoplastic nasal bone, upper limb defects, small ventricular septal defect, enlarged, echogenic and cystic right kidney in fetus on ultrasonography. Fetal anthropometry was within normal limits. Fetus had facial asymmetry, hypertelorism, short nose, anteverted nares, long philtrum, retrognathia, bilateral radial deviation of hands with bowed forearms, rudimentary right thumb and absence of two digits (ray defect - F1 and F2) on left hand. Female external genitalia was noted with no perineal openings and presence of single umbilical artery.

Membranous ventricular septal defect, unilateral (left) renal agenesis with absent left ureter, normal right kidney, ?ovaries, no uterine tissue, blind-ending persistent cloaca with right ureter and hindgut draining into it were documented on visceral examination. Infantogram showed absent radius on left side and rudimentary radius on right side, absence of metacarpals and phalanges of first and second digits on left hand and absent metacarpal and phalanges of right thumb (Supplementary figure 2).

**Fetus 6 (Thoraco-abdominoschisis, limb defects and urorectal septum malformation sequence)**

**Pedigree:**


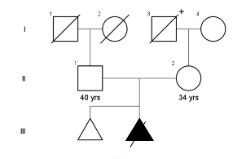


14 weeks

**
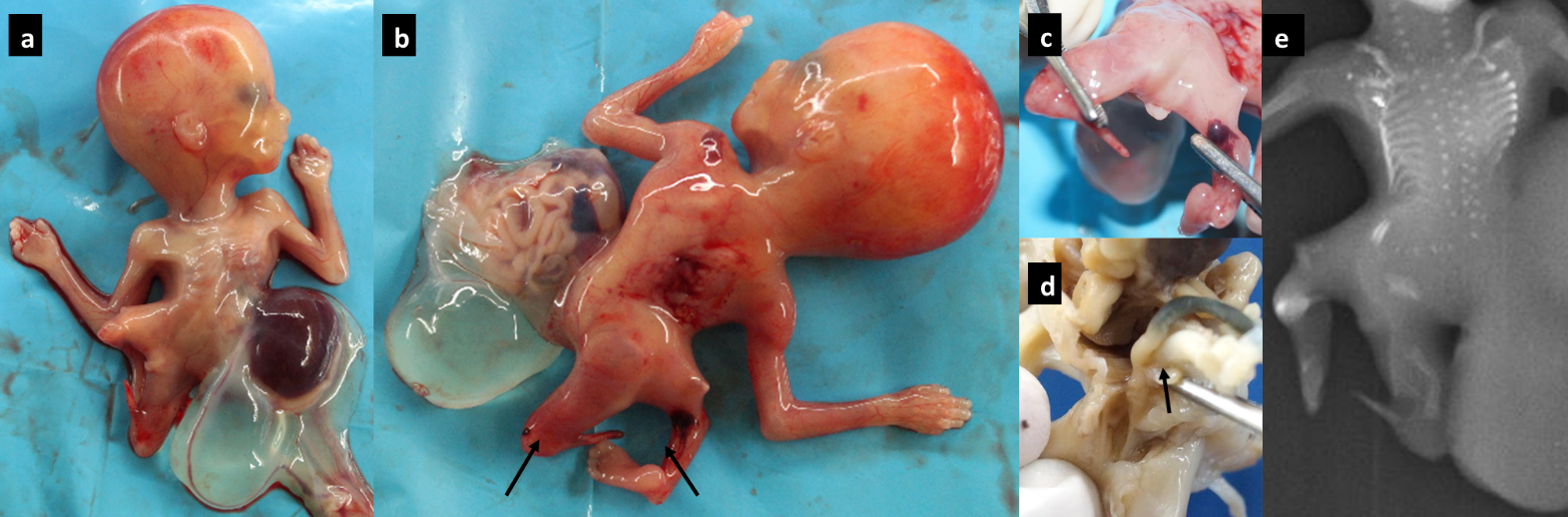
Supplementary figure 3:** 14 weeks fetus (6) had anterior lower thoracic and abdominal wall defect with herniation of liver and intestine, kypho-scoliosis (a, b). Short right lower limb with contractures and reduction defect of left lower limb below the knee (arrows), ambiguous genitalia with absent perineal openings (b, c), absent left kidney and adrenal with blind ending persistent cloaca (arrow, d) were also noted. Radiographs show kypho-scoliosis at thoraco-lumbar region and absence of ossification of lower lumbar and sacral vertebrae and bent left femur (arrow) and underdeveloped long bones of right lower limb (e).

**Clinical features:** The pregnancy was interrupted at 14 weeks of gestation with anterior abdominal wall defect and herniation of liver in fetus. On evaluation, fetus measured 8.4 cm (-2.9 SD) in total length with anterior lower thoracic and abdominal wall defect, herniation of stomach, spleen, pancreas, liver and loops of small intestine covered by peritoneum, kypho-scoliosis at thoraco-lumbar region, short right lower limb with joint contractures across hips, knees and ankles with bowing of thighs and legs, transverse deficiency of left lower limb below the knee with rudimentary stem like structure (Supplementary figure 3). There was imperforate anus with ambiguous external genitalia.

On internal examination, we note dextrocardia with a defect in lower portion of sternum through which the portion of left lung and apex of heart protruded into the abdominal cavity with absent left dome of diaphragm. Agenesis of left kidney and adrenal gland was noted. There was urorectal septum malformation sequence with persistent cloaca. The colon and right ureter drained into cloaca with atresia of cloacal outlet. Indifferent gonads, kypho-scoliosis at thoraco-lumbar region, absence of ossification of lower lumbar and sacral vertebrae, bowed left femur, short and bowed right femur, short right tibia and absence of right fibula were observed.

**Fetus 7 (Myelomeningocele, urorectal septum malformation sequence, bilateral renal agenesis and vertebral segmentation defect)**

**Pedigree:**

**
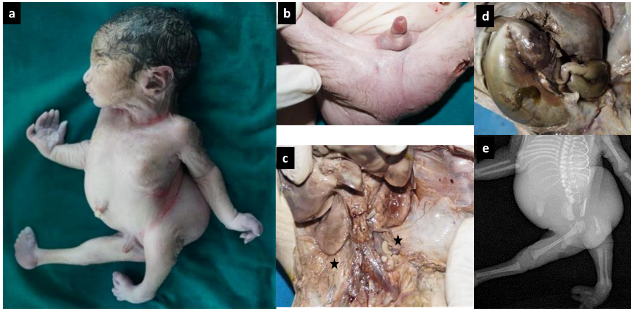
**

**Supplementary figure 4:** Evaluation of fetus 7 revealed dysmorphic ears, short trunk, scoliosis, unilateral radial deviation of right hand, contractures across axillae, elbows, hips and knees joints, bilateral clubfeet and rhizomelia of left lower limb (a), ambiguous external genitalia with no perineal openings (b), bilateral renal agenesis (c, asterisks), meconium filled hindgut (d) and segmentation defects of thoracic and lumbar vertebrae, absent ossification of sacral vertebrae and short left femur (e).

**Clinical features:** A stillbirth was referred for detailed examination at 36 weeks of gestation following ultrasound findings of intrauterine growth retardation, large open neural defect at lower half of spine and anhydramnios. Anthropometry suggested [weighed 1131 g (-3SD to -4SD) measured 33 cm (-4SD) in length with head circumference of 27.5 cm (-2SD to -3SD)] fetal growth retardation. Dysmorphic ears, short trunk, scoliosis at lumbar region, lipomeningomyelocele, unilateral radial club hand on right side, bilateral clubfeet and rhizomelia of left lower limb, bilateral contractures across axilla, elbow, hip and knee were evident in the fetus. Ambiguous external genitalia with phallus-like structure and absent perineal openings were observed.

There were pulmonary hypoplasia, bilateral renal agenesis with absent ureters, short small intestine, enlarged hind gut filled with feces draining into the blind cloaca with no intra-abdominal gonads in fetus (Supplementary figure 4). Segmentation defects of thoracic and lumbar vertebrae, absent ossification of sacral vertebrae, short left femur and herniation of dural sac at sacral region with spinal cord were reported on fetal imaging.

**Fetus 8 (Urorectal septal malformation sequence and neural tube defect)**

**
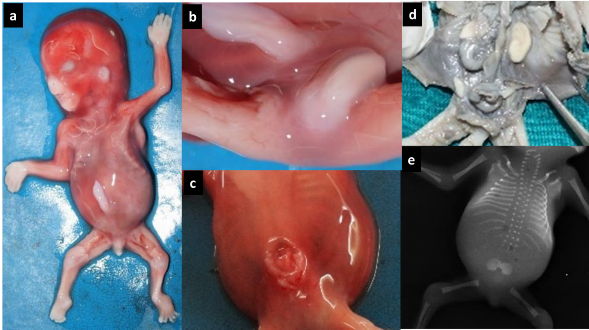
**

**Supplementary figure 5**: Intrauterine demise of fetus 8 was noted with phallus-like structure (asterisk) with no perineal openings (a, b), spina bifida at lower lumbar region (c), left renal agenesis with blind ending cloaca (black arrow, d) and fused iliac bones (white arrow) and absence of ossification for lower lumbar vertebrae (e).

**Clinical features:** The fetus was examined following intrauterine demise and with an umbilical cord cyst. The fetus had open neural tube defect at lower lumbar region, ambiguous external genitalia with phallus-like structure and imperforate anus. Unilateral (left) renal agenesis was noted with right ureter and hindgut opening into blind cloaca. There were indifferent gonads. Radiographs of the fetus revealed fused iliac bones and absence of ossification of lower lumbar vertebrae (Supplementary figure 5).

**Fetus 9 (Omphalocele, partial urorectal septum malformation, imperforated anus and scoliosis)**

**Pedigree:**

**
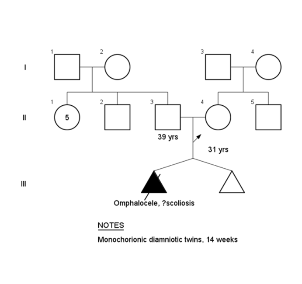
**

**
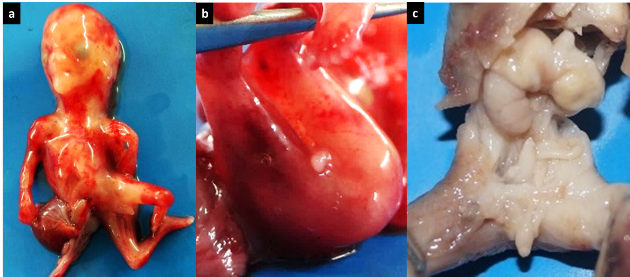
**

**Supplementary figure 6**: Omphalocele, scoliosis (a), ambiguous external genitalia with no perineal openings (arrow, b) and partial separation of urogenital sinus and primitive rectum (arrow, c) were observed in fetus 9.

**Clinical features:** A twin from monochorionic diamniotic pregnancy weighed 20g (-2SD to -3SD) and measured 9 cm (-2SD to -3SD) in length at 14 weeks of gestation was evaluated in view of omphalocele and scoliosis. Postnatal examination of fetus confirmed omphalocele and scoliosis at lumbar region. In addition, ambiguous external genitalia with phallus-like structure, imperforate anus and unilateral club foot on left side were noted (Supplementary figure 6). There was partial separation of urogenital sinus and primitive rectum in fetus.

**Fetus 10 (Occipital encephalocele, renal agenesis and urorectal septum malformation sequence)**

**Pedigree:**

**
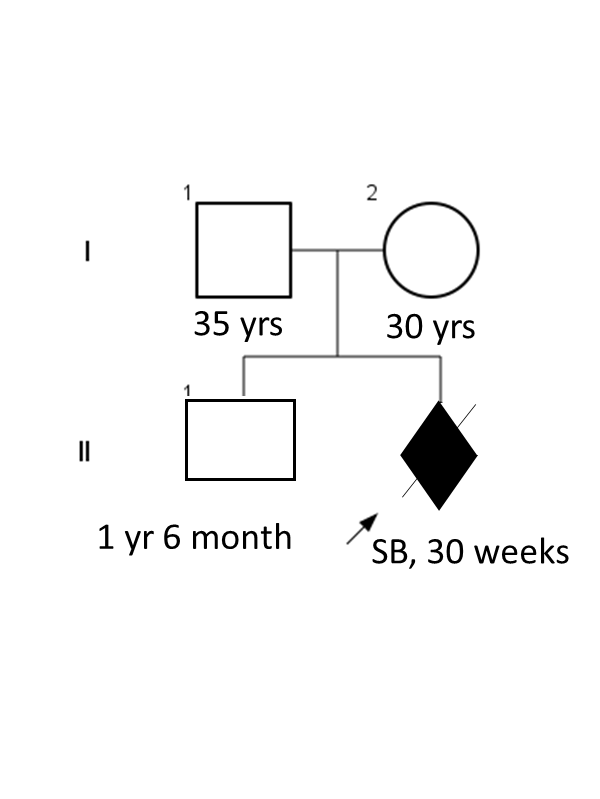
**

**
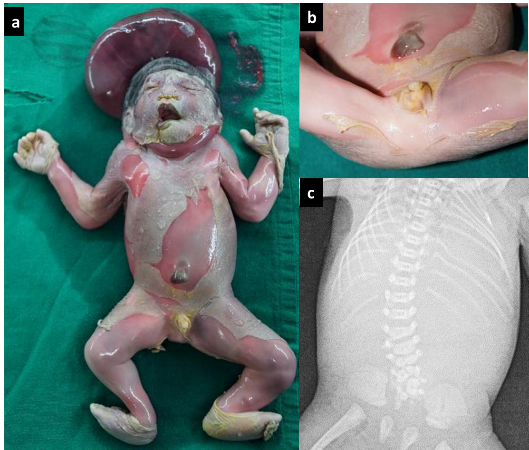
**

**Supplementary figure 7:** Occipital encephalocele (asterisk, a), small phallus-like structure with absent perineal openings (a, b) and segmentation defects of lumbar vertebrae (arrow, c) were noted in 30 weeks’ fetus (10).

**Clinical features:** Stillbirth was noted at 30 weeks of gestation. Imaging findings of occipital encephalocele, bilateral echogenic kidneys and anhydramnios were noted antenatally. The fetus weighed 718 g (-2SD to -3SD) measured 29 cm (-5SD) in total length with frontal sloping, flat nasal tip, micrognathia, dysmorphic ears, short neck and occipital encephalocele (Supplementary figure 7). Ambiguous external genitalia was noted with no perineal openings. Bilateral pulmonary hypoplasia was observed. There were bilateral renal agenesis with absent ureters, the rudimentary bladder and hindgut draining into a blind, persistent cloaca. Indistinct gonads were noted with persistent left mesonephric cord. Radiographs of the fetus showed segmentation defect in lower lumbar vertebrae and absence of ossification of sacrum.
